# The “echo-free space” technique: A safe and reliable method for endoscopic ultrasound scope insertion into the esophagus

**DOI:** 10.1055/a-2325-2339

**Published:** 2024-06-12

**Authors:** Shunsuke Omoto, Mamoru Takenaka, Kota Takashima, Yoriaki Komeda, Masatoshi Kudo

**Affiliations:** 1 Department of Gastroenterology and Hepatology, Kindai University Faculty of Medicine, Osaka-Sayama, Japan

In recent years, the use of endoscopic ultrasound-guided fine-needle aspiration (EUS-FNA) and EUS-guided drainage has greatly expanded 1 2 3 4 . However, inserting a linear EUS scope into the esophagus can be challenging, particularly in intubated or pediatric patients. This is because the scope is side-viewing, and the tip does not face the cervical esophagus. As a result, it can damage the laryngopharynx and lead to unsuccessful insertion if advanced, based on endoscopic imaging ( Fig. 1 ).

**Fig. 1:**
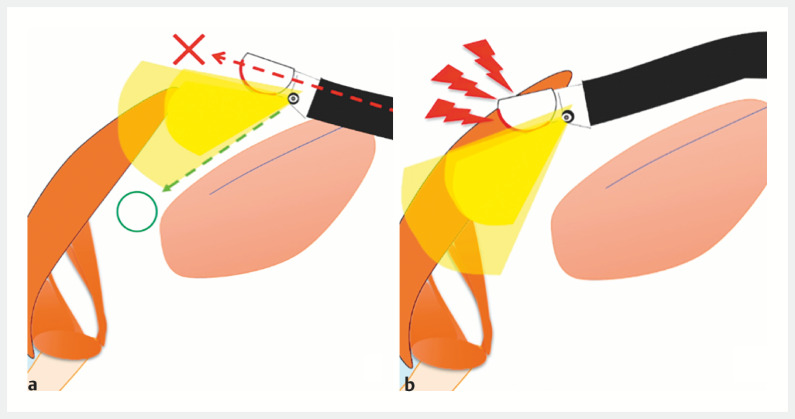
**a** The tip of the side-viewing scope does not face the cervical esophagus. **b** It can damage the laryngopharynx and lead to unsuccessful insertion if advanced, based on endoscopic imaging.

Because of these difficulties, inserting an EUS scope into the esophagus can be a hurdle for trainees learning this procedure. Even expert EUS sonographers may experience difficulties, resulting in repeated insertion attempts and potential damage to the laryngopharynx.

We present the case of an 8-year-old girl with an infected pancreatic pseudocyst who underwent emergency EUS-guided drainage under general anesthesia and tracheal intubation. Initially, scope insertion into the esophagus was difficult due to contact with the intubation tube ( Fig. 2 ). In this situation, the technique of inserting the EUS scope safely while confirming the lumen of the esophagus was required. In this case, we used the “echo-free space” technique, which is a safe insertion technique for EUS scopes that depicts the digestive tract lumen as echo-free space 5 .

**Fig. 2:**
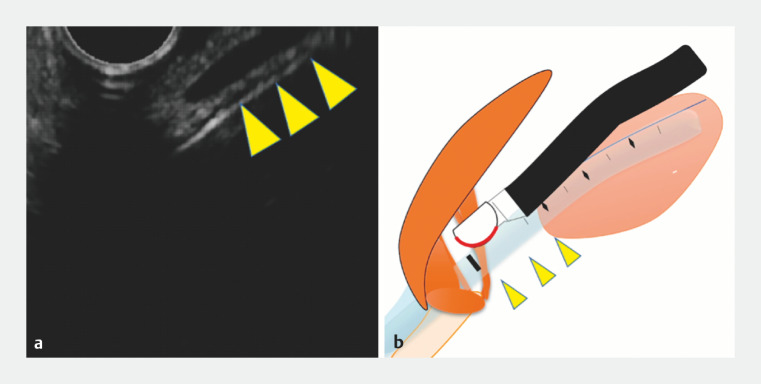
The insertion of the scope into the esophagus was difficult due to contact with the intubation tube (arrowhead).

First, the cervical esophageal lumen, which is depicted by EUS as an echo-free space, was sought and successfully identified ( Fig. 3 ). Then, the tip of the scope was advanced toward the echo-free space ( Fig. 4 ). By aligning the tip of the scope with the echo-free space on the EUS image, successful insertion of the EUS scope into the esophagus was achieved ( Video 1 ).

**Fig. 3:**
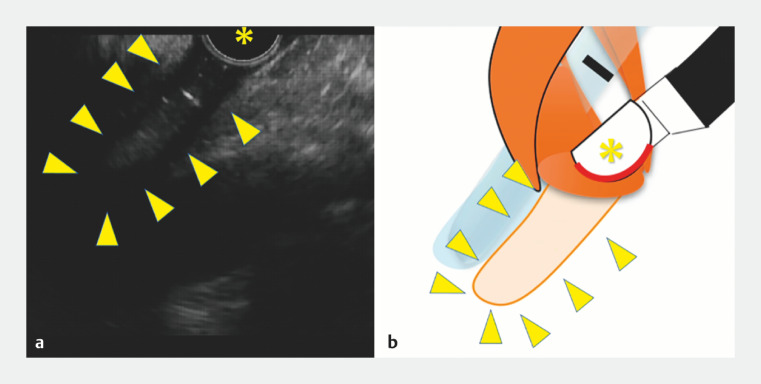
The cervical esophageal lumen, which was depicted by EUS as an "echo-free space," was successfully identified (arrowhead).

**Fig. 4:**
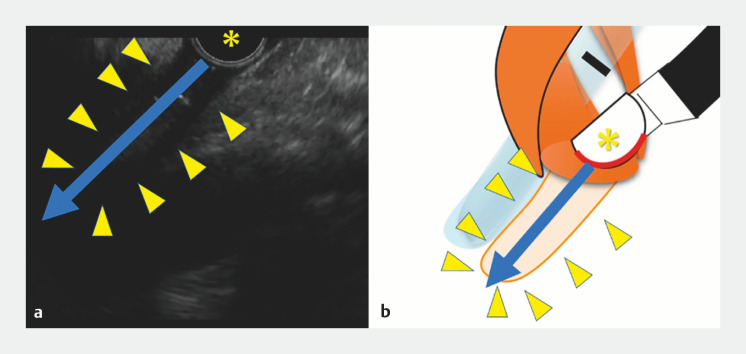
The tip of the scope (*) was advanced toward echo-free space (arrowhead). By aligning the tip of the scope with the echo-free space on the EUS image, successful insertion of the EUS scope into the esophagus was achieved.

This video introduces the “echo-free space” method, which allows safe insertion of the EUS scope while confirming the esophageal lumen as the echo-free space.Video 1

This echo-free space technique, which is the safe EUS scope insertion technique guided by an EUS image, can be helpful for trainees and experts when esophageal insertion of the EUS scope is difficult.

Endoscopy_UCTN_Code_TTT_1AS_2AB

## Notes

Endoscopy E-Videos https://eref.thieme.de/e-videos
*E-Videos* is an open access online section of the journal *Endoscopy* , reporting on interesting cases and new techniques in gastroenterological endoscopy. All papers include a high-quality video and are published with a Creative Commons CC-BY license. Endoscopy E-Videos qualify for HINARI discounts and waivers and eligibility is automatically checked during the submission process. We grant 100% waivers to articles whose corresponding authors are based in Group A countries and 50% waivers to those who are based in Group B countries as classified by Research4Life (see: https://www.research4life.org/access/eligibility/ ). This section has its own submission website at https://mc.manuscriptcentral.com/e-videos .
